# Outdoor play and catch-up growth in small-for-gestational-age children: a nationwide prospective birth cohort study from the Japan environment and children’s study

**DOI:** 10.1186/s12889-026-28796-x

**Published:** 2026-08-03

**Authors:** Yoshinori Aoki, Michikazu Nakai, Hirotake Sawada, Hiroshi Moritake, the Japan Environment and Children’s Study Group, Michihiro Kamijima, Shin Yamazaki, Maki Fukami, Reiko Kishi, Chiharu Ota, Koichi Hashimoto, Kenichi Sakurai, Shuichi Ito, Ryoji Shinohara, Hidekuni Inadera, Takeo Nakayama, Ryo Kawasaki, Yasuhiro Takeshima, Hideki Nagashima, Narufumi Suganuma, Mayumi Tsuji, Kimitoshi Nakamura

**Affiliations:** 1 Division of Pediatrics, Faculty of Medicine, University of Miyazaki, 5200 Kiyotakecho Kihara, Miyazaki, Japan; 2 Department of Statistics and Data Management, Faculty of Medicine, University of Miyazaki, Miyazaki, Japan

**Keywords:** Small for gestational age, Catch-up growth, Outdoor play, Birth cohort study, Physical activity, Child growth

## Abstract

**Background:**

Children born small for gestational age (SGA) are at increased risk of impaired growth and adverse long-term health outcomes. While several prenatal and perinatal determinants of catch-up growth have been identified, evidence regarding modifiable postnatal behavioral factors remains limited. Outdoor play, which reflects both physical activity and environmental exposure, may influence linear growth during early childhood. This study aimed to examine the association between outdoor play and catch-up growth by age 3 years among children born SGA.

**Methods:**

This nationwide prospective birth cohort study used data from the Japan Environment and Children’s Study (JECS). Among 104,043 registered fetuses, 1,796 children born SGA with available data on outdoor play and height at age 3 years were included. Caregivers reported children’s outdoor playtime during summer and winter between ages 1–2 and 2–3 years. Outdoor play was categorized as no outdoor play in either season, outdoor play in one season, or outdoor play in both seasons. The primary outcome was catch-up growth, defined as height ≥ − 2 standard deviations at age 3 years. Multivariable logistic regression models were used to estimate adjusted odds ratios (AORs) and 95% confidence intervals (CIs), adjusting for maternal, perinatal, and environmental factors.

**Results:**

Among 1,796 children born SGA, 296 (16.5%) did not achieve catch-up growth by age 3 years. No outdoor play in either season between ages 1–2 years was associated with higher odds of failure to achieve catch-up growth compared with outdoor play in both seasons (AOR 2.51; 95% CI, 1.22–5.18). A similar association was observed between ages 2–3 years (AOR 4.36; 95% CI, 1.42–13.38). Outdoor play in only one season was not significantly associated with catch-up growth.

**Conclusions:**

Outdoor play during early childhood was associated with catch-up growth by age 3 years among children born SGA. While these findings suggest that outdoor play may represent a potentially modifiable behavioral factor related to early growth, the observational nature of the study precludes causal inference. Further studies are needed to clarify underlying mechanisms and to determine whether promoting outdoor play can support healthy linear growth in this population.

**Supplementary Information:**

The online version contains supplementary material available at https://doi.org/10.1186/s12889-026-28796-x.

## Background

Small for gestational age (SGA) refersto neonates born with a birth weight and/or length below the normal range for their gestational age [1, 2]. Although definitions of SGA vary, it is estimated that approximately 16% of all births worldwide are classified as SGA, ranging from 7% in high-income countries to 40% in South Asia [3]. Infants born SGA have a higher mortality rate than those born appropriate for gestational age [4]. In addition, children born SGA are at increased risk of adverse neurodevelopmental and psychological outcomes later in life, including developmental delays, impaired cognition, and attention-deficit/hyperactivity disorder [1, 5]. They are also at increased risk of metabolic disorders and obesity from childhood through adolescence [1, 6].

Catch-up growth during early childhood is a key determinant of long-term health in children born SGA. Approximately 85%–90% of infants born SGA achieve catch-up growth in height by the age of 3 years [1, 7]. However, excessively rapid catch-up growth has been associated with an increased risk of later obesity and cardiometabolic disease [8]. In contrast, failure to achieve catch-up growth increases the risk of short stature in later life [2]. Children who do not exhibit catch-up growth are at a higher risk of health-related problems, developmental delays, and behavioral disorders [6, 9]. Therefore, identifying modifiable determinants of healthy linear growth in this population is an important public health priority.

Previous research has identified several prenatal and perinatal factors associated with catch-up growth in children born SGA. Among prenatal factors, high maternal total cholesterol has been associated with failure to achieve catch-up growth in full-term infants born SGA [10]. In addition, elevated levels of cadmium in cord blood have been associated with failure to achieve catch-up growth [11]. Regarding birth conditions, lower gestational age and a lower height z-score at birth may reduce the likelihood of achieving subsequent catch-up growth [12, 13]. Associations with genetic and epigenetic factors have also been reported [14].

In contrast, evidence on postnatal environmental and behavioral factors related to catch-up growth remains limited. Breastfeeding has been suggested to support favorable growth and body composition [2], yet other modifiable lifestyle factors—particularly those related to physical activity and outdoor behaviors—remain poorly understood. Outdoor play is a common behavior in early childhood that reflects both physical activity and environmental exposure and may influence linear growth.

However, few studies have examined the association between outdoor play or other physical activity and growth outcomes specifically in children born SGA. Prior research has largely focused on physical fitness or quality of life in adulthood [15]. To our knowledge, no study has directly evaluated the association between outdoor play during early childhood and catch-up growth in children born SGA. Given concerns about declining outdoor activity among young children worldwide [16], clarifying its role in early growth may have clinical and public health implications.

In the present study, we examined the association between outdoor play in early childhood and catch-up growth at the age of 3 years in children born SGA, using data from a nationwide birth cohort in Japan. We hypothesized that a lack of outdoor play, particularly across seasons, would be associated with failure to achieve catch-up growth.

## Methods

### Study design and setting

This study analyzed data from the Japan Environment and Children’s Study (JECS), an ongoing nationwide prospective birth cohort study designed to assess the influence of environmental, medical, psychosocial, and lifestyle factors during pregnancy and childhood on child health and development. The JECS was initiated in 2011 as a collaboration among the Programme Office (National Institute for Environmental Studies), the Medical Support Centre (National Center for Child Health and Development), and 15 Regional Centres across Japan. Pregnant women were recruited between January 2011 and March 2014 through Co-operating Health Care Providers and local government offices.

The study protocol was reviewed and approved by the Ministry of the Environment’s Institutional Review Board on Epidemiological Studies and the Ethics Committees of all participating institutions (Ethical No. 100910001). The study was conducted in accordance with the Declaration of Helsinki. All participants provided written informed consent. This manuscript was prepared in accordance with the Strengthening the Reporting of Observational Studies in Epidemiology (STROBE) guidelines. Further details regarding the JECS methodology have been reported previously [17, 18].

### Participants

A total of 104,043 fetuses were registered in the JECS cohort. Participants were eligible if they resided in one of the designated Study Areas; had an expected delivery date between August 1, 2011 and mid-2014; planned to remain in Japan long-term; and had sufficient Japanese proficiency to complete a self-administered questionnaire.

This analysis used the dataset released in March 2025. Among 104,043 fetuses, 100,112 live births were included. We excluded cases with unknown sex; unknown gestational age or a gestational age < 22 weeks or ≥ 42 weeks at birth; missing data on birth length or weight; unknown parity; and those with congenital anomalies. SGA was defined as a birth length and/or weight < − 2 SD according to the international consensus guideline [2]. Z-scores for birth length and birth weight by gestational age were calculated based on Japanese neonatal anthropometric charts [19]. Data on gestational age and neonatal anthropometric measurements were transcribed from the medical records.

Among the eligible infants, 2,509 were classified as SGA. We further excluded those with missing height data at the age of 3 years or missing information on outdoor playtime between the ages of 2 and 3 years. The final analysis sample consisted of 1,796 children born SGA.

### Outcome

The primary outcome was catch-up growth, defined as a height at the age of 3 years ≥ − 2 SD for age, based on previous studies [7, 9, 13]. The age of 3 years was chosen as a reference point because previous studies on children born SGA indicated that the rate of catch-up growth does not markedly increase beyond this age [12, 20]. Height at the age of 3 years was reported by guardians via a self-administered questionnaire. Measurements were primarily taken at medical institutions, municipal facilities, or childcare facilities. The z-score for height was calculated using Japanese growth standard charts [21].

### Exposure

Outdoor playtime between the ages of 1 and 2 years and 2 and 3 years was reported by caregivers and categorized separately for summer (May to September) and winter (November to February). Caregivers selected one of the following five options: “hardly plays outside,” “less than 1 h per day,” “1 to < 3 h per day,” “ ≥ 3 h per day,” or “unknown.” Responses marked as “unknown” were excluded from the analysis.

Outdoor playtime was then dichotomized as “no” (hardly plays outside) or “yes” (all other categories). This dichotomization was chosen to specifically contrast children with no outdoor play against those with any exposure, as children with no outdoor play may represent a high-risk group with minimal exposure to physical activity and sunlight, both of which are hypothesized to influence growth. This contrast was also considered relevant from a public health perspective.

Based on this classification, children were further grouped into three categories for the main analysis: “no outdoor play in either season,” “outdoor play in either summer or winter,” and “outdoor play in both seasons.” This procedure was applied separately for outdoor playtime between the ages of 1 and 2 years and 2 and 3 years. We examined potential interaction between summer and winter outdoor play; however, no statistically significant interaction was observed. Therefore, we did not further stratify the analyses by season.

We also examined the consistency of outdoor play patterns between the two time periods using cross-tabulation and Pearson’s chi-square test.

### Covariates

Covariates were selected a priori based on prior literature, biological plausibility, and a conceptual framework of factors potentially associated with both outdoor play and postnatal growth among children born SGA [13, 22–26]. A variable was considered a potential confounder if it was associated with both the exposure and the outcome, regardless of its direct association with SGA status. The following variables were included in the analysis: maternal age at delivery, maternal height, maternal educational attainment, paternal educational attainment, household income, child’s sex, gestational age, birth length z-score, feeding type at 6 months of age (breastfeeding, formula feeding, or mixed feeding), household smoking exposure, and postnatal health conditions that may influence both physical activity and growth (including malignancy, cerebral palsy, and bronchial asthma).

### Statistical analysis

Continuous variables are presented as medians with interquartile ranges (IQRs), and categorical variables are expressed as numbers with percentages. Univariate and multivariable logistic regression analyses were performed to examine the association between outdoor play and catch-up growth, with separate models constructed for outdoor play between the ages of 1 and 2 years and 2 and 3 years. The covariates described above were included in all multivariable models. No imputation was performed for missing data, and analyses were performed using a complete-case approach. We additionally performed sex-stratified analyses as exploratory analyses and formally tested for interaction by sex. We also conducted stratified analyses by gestational age (term vs. preterm) and a sensitivity analysis restricted to singleton children. To assess potential selection bias due to missing data, we compared baseline characteristics between participants included in the analysis and those excluded because of missing information (Additional file 1: Supplementary eTable 1). All analyses were performed using Stata ver. 19 (StataCorp, College Station, TX, USA). A two-sided *p* value of < 0.05 was considered statistically significant.

## Results

**Fig. 1:**
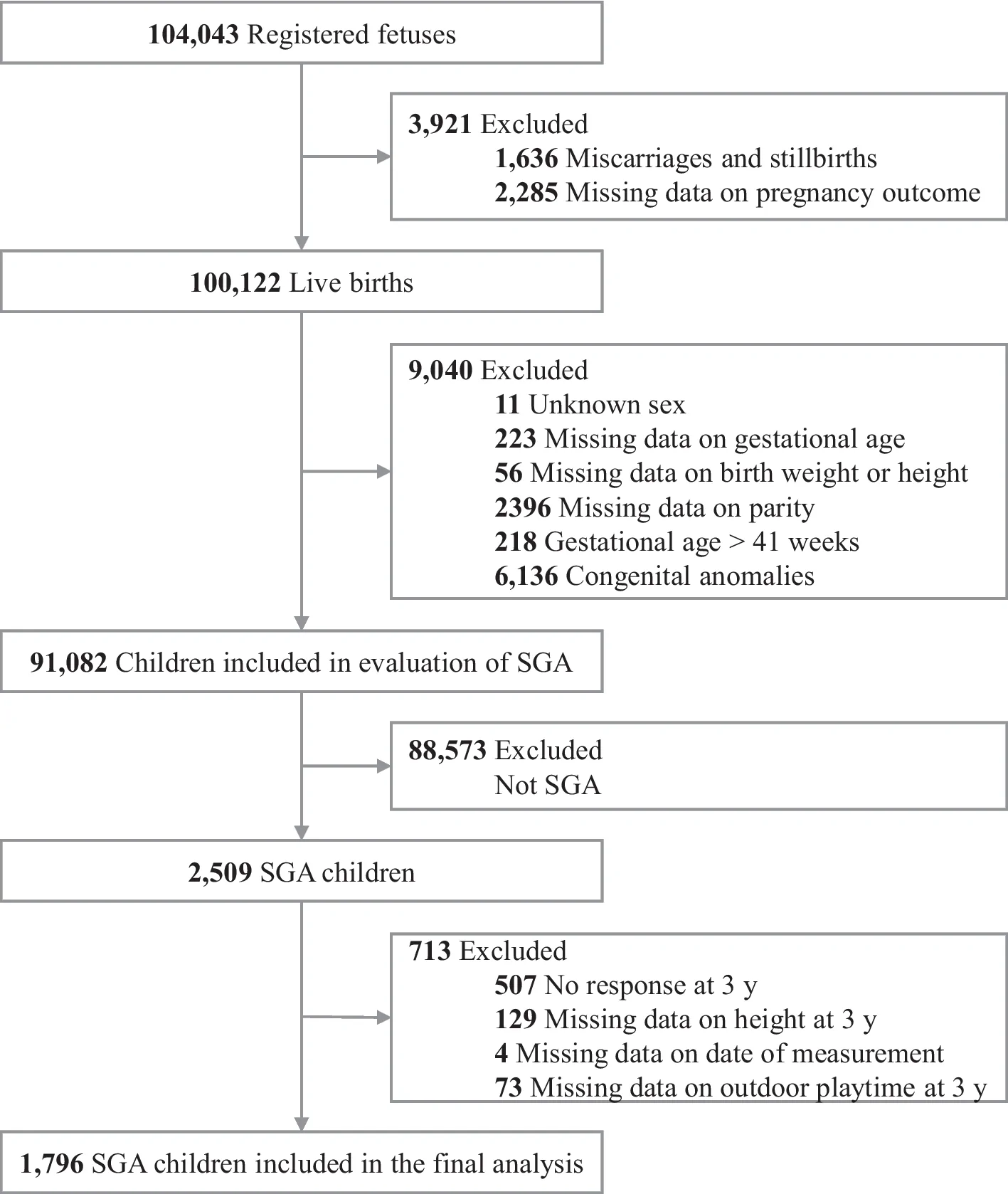
Flowchart of participant selection. This figure illustrates the selection process of children born small for gestational age (SGA) included in the final analytic sample from the Japan Environment and Children’s Study. Participants were excluded because of missing perinatal data, congenital anomalies, missing height measurements at age 3 years, or missing data on outdoor playtime. Abbreviation: SGA, small for gestational age

Figure 1 shows the flowchart of participant selection. Among 104,043 registered fetuses in the JECS, 1,796 children born SGA met the eligibility criteria and had complete data on both outdoor play and height at the age of 3 years. A comparison of baseline characteristics between included and excluded participants is presented in Supplementary eTable 1. Several differences were observed in maternal and socioeconomic characteristics, including maternal age, maternal height, parity, parental educational attainment, and household income, as well as postnatal health conditions. In contrast, no clear differences were observed in outdoor play, the main exposure of interest; however, a substantial proportion of excluded participants had missing data for this variable, particularly at ages 2–3 years.

Table 1 summarizes the characteristics of the study population. The median gestational age at birth was 39 weeks (IQR, 38–39 weeks), and 220 children (12.2%) were preterm (< 37 weeks of gestation). The median birth length z-score was − 2.1 (IQR, − 2.4 to − 1.4), and the median birth weight z-score was − 2.1 (IQR, − 2.4 to − 1.6). Boys accounted for 42.0% (*n* = 755) of the sample, and girls for 58.0% (*n* = 1,041). Among the 1,796 participants, 296 (16.5%) had failure to achieve catch-up growth by the age of 3 years. Baseline characteristics according to catch-up growth status are shown in Supplementary eTable 2, with some differences observed across demographic and clinical variables. Outdoor play patterns were largely consistent across the two time points; 1306/1702 (76.3%) of children remained in the same outdoor play category between ages 1–2 and 2–3 years (*p* < 0.001; see eTable 3 for details).

**Table 1:** Characteristics of study participants

Characteristic	Total (*n* = 1,796)	Participants	
		Boy (*n* = 755)	Girl (*n* = 1,041)
Maternal age at delivery, median (IQR), y	31.0 (28.0, 35.0)	31.0 (28.0, 35.0)	31.0 (28.0, 35.0)
Maternal height, median (IQR), cm	157.0 (153.0, 160.0)	157.0 (153.0, 160.0)	156.1 (153.0, 160.0)
Maternal smoking during pregnancy			
No	1,661 (92.5%)	703 (93.%)	958 (92%)
Yes	115 (6.4%)	44 (5.8%)	71 (6.8%)
Missing data	20 (1.1%)	8 (1.1%)	12 (1.2%)
Parity			
0	935 (52.1%)	386 (51.1%)	549 (52.7%)
≥ 1	861 (47.9%)	369 (48.9%)	492 (47.3%)
Plurality			
Singleton	1,695 (94.4%)	703 (93.1%)	992 (95.3%)
Mutiple	101 (5.6%)	52 (6.9%)	49 (4.7)
Mother educational level			
Junior high school	85 (4.7%)	32 (4.2%)	53 (5.1%)
High school	571 (31.8%)	235 (31.1%)	336 (32.3%)
Technical college, Vocational school, Junior college	739 (41.1%)	328 (43.4%)	411 (39.5%)
University/College, Graduate school	373 (20.8%)	145 (19.2%)	228 (21.9%)
Missing data	28 (1.6%)	15 (2.0%)	13 (1.2%)
Father educational level			
Junior high school	126 (7.0%)	54 (7.2%)	72 (6.9%)
High school	639 (35.6%)	261 (34.6%)	378 (36.3%)
Technical college, Vocational school, Junior college	432 (24.1%)	192 (25.4%)	240 (23.1%)
University/College, Graduate school	562 (31.3%)	229 (30.3%)	333 (32.0%)
Missing data	37 (2.1%)	19 (2.5%)	18 (1.7%)
Household annual income (JPY)			
< 2,000,000	85 (4.7%)	34 (4.5%)	51 (4.9%)
2,000,000–3,999,999	607 (33.8%)	262 (34.7%)	345 (33.1%)
4,000,000–5,999,999	537 (29.9%)	227 (30.1%)	310 (29.8%)
6,000,000–7,999,999	257 (14.3%)	101 (13.4%)	156 (15.0%)
8,000,000–9,999,999	122 (6.8%)	53 (7.0%)	69 (6.6%)
10,000,000–11,999,999	35 (1.9%)	17 (2.3%)	18 (1.7%)
12,000,000–14,999,999	13 (0.7%)	5 (0.7%)	8 (0.8%)
15,000,000–19,999,999	9 (0.5%)	3 (0.4%)	6 (0.6%)
≥ 20,000,000	5 (0.3%)	2 (0.3%)	3 (0.3%)
Missing data	126 (7.0%)	51 (6.8%)	75 (7.2%)
Gestational age, median (IQR), wk	39.0 (38.0, 40.0)	39.0 (38.0, 40.0)	39.0 (38.0, 40.0)
Preterm			
No	1,576 (87.8%)	644 (85.3%)	932 (89.5%)
Yes	220 (12.2%)	111 (14.7%)	109 (10.5%)
Birth length, median (IQR), cm	45.0 (43.6, 46.0)	45.1 (43.8, 47.0)	45.0 (43.6, 46.0)
Birth length z score, median (IQR)	−2.1 (−2.4, −1.5)	−2.1 (−2.3, −1.2)	−2.2 (−2.4, −1.8)
Birth weight, median (IQR), g	2372 (2126, 2575)	2350 (2076, 2540)	2390 (2152, 2588)
Birth weight z score, median (IQR)	−2.1 (−2.4, −1.5)	−2.1 (−2.4, −1.8)	−2.0 (−2.3, −1.3)
Feeding at 6 months of age			
Breast milk only	570 (31.7%)	239 (31.7%)	331 (31.8%)
Formula milk only	49 (2.7%)	15 (2.0%)	34 (3.3%)
Mixed	1147 (63.9%)	487 (64.5%)	660 (63.4%)
Missing data	30 (1.7%)	14 (1.9%)	16 (1.5%)
Household smoking exposure			
No	1,071 (59.6%)	459 (60.8%)	612 (58.8%)
Yes	725 (40.4%)	296 (39.2%)	429 (41.2%)
Postnatal health conditions	179 (10.0%)	107 (14.2%)	72 (6.9%)
Outdoor play (1–2 years)			
Outdoor play in both summer and winter	1,381 (76.9%)	583 (77.2%)	798 (76.7%)
Outdoor play in either summer or winter	277 (15.4%)	108 (14.3%)	169 (16.2%)
No outdoor play in either summer or winter	54 (3.0%)	25 (3.3%)	29 (2.8%)
Missing data	84 (4.7%)	39 (5.2%)	45 (4.3%)
Outdoor play (2–3 years)			
Outdoor play in both summer and winter	1,588 (88.4%)	672 (89.0%)	916 (88.0%)
Outdoor play in either summer or winter	181 (10.1%)	73 (9.7%)	108 (10.4%)
No outdoor play in either summer or winter	27 (1.5%)	10 (1.3%)	17 (1.6%)

Values are presented as number (percentage) for categorical variables and median (interquartile range) for continuous variables. SGA was defined as birth weight below the 10th percentile for gestational age. Catch-up growth was defined as achieving a height above − 2 standard deviation scores at age 3 years. Differences between groups were assessed using the chi-square test for categorical variables and the Mann–Whitney U test for continuous variables

*Abbreviations*: *h* Hours, *IQR* Interquartile range, *JPY* Japanese yen, *SGA* Small for gestational age, *y* Years

The associations between outdoor play and failure to achieve catch-up growth are shown in Table 2. A lack of outdoor play in both summer and winter between the ages of 1 and 2 years and 2 and 3 years was associated with a higher likelihood of failure to achieve catch-up growth at the age of 3 years.

**Table 2:** Associations between outdoor play and catch-up growth at age 3 years in children born SGA

Exposure	Not achieving catch-up growth			
	Univariate		Multivariable	
	OR (95% CI)	***p*** value	AOR (95% CI)	***p*** value
1–2 years				
Outdoor play in both summer and winter	1 [Reference]		1 [Reference]	
Outdoor play in either summer or winter	0.87 (0.61–1.25)	0.464	0.95 (0.60–1.44)	0.747
No outdoor play in either summer or winter	2.31 (1.28–4.18)	0.006^a^	2.51 (1.22–5.18)	0.013^a^
2–3 years				
Outdoor play in both summer and winter	1 [Reference]		1 [Reference]	
Outdoor play in either summer or winter	1.17 (0.78–1.74)	0.453	0.96 (0.57–1.60)	0.863
No outdoor play in either summer or winter	2.20 (0.95–5.08)	0.065	4.36 (1.42–13.38)	0.010^a^

Odds ratios (ORs) and 95% confidence intervals (CIs) were estimated using logistic regression models

*Abbreviations*: *AOR* Adjusted odds ratio, *CI* Confidence interval, *OR* Odds ratio

^a^*p* < 0.05

Between the ages of 1 and 2 years, children who did not play outdoors in either summer or winter had higher odds of failure to achieve catch-up growth compared with those who played outdoors in both seasons (OR, 2.31; 95% CI, 1.28–4.18; *p* = 0.006). After multivariable adjustment, this association remained statistically significant (AOR, 2.51; 95% CI, 1.22–5.18; *p* = 0.013). Children who played outdoors in only summer or only winter did not show a statistically significant association with failure to achieve catch-up growth compared with those who played outdoors in both seasons (AOR, 0.93; 95% CI, 0.60–1.44).

A similar pattern was observed for outdoor play reported between the ages of 2 and 3 years. In the univariate analysis, children who hardly played outdoors in either season had higher odds of failure to achieve catch-up growth, although this did not reach statistical significance (univariate OR, 2.20; 95% CI, 0.95–5.08; *p* = 0.065). After multivariable adjustment, the association became statistically significant (AOR, 4.36; 95% CI, 1.42–13.38;* p* = 0.010). As observed between the ages of 1 and 2 years, outdoor play in only one season was not significantly associated with catch-up growth outcomes (AOR, 0.96; 95% CI, 0.57–1.60). The original dataset used for the analysis is summarized in eTable 4.

Sex-stratified analyses are presented in Table 3. Among boys, a lack of outdoor play in both summer and winter between the ages of 1 and 2 years was associated with a higher likelihood of failure to achieve catch-up growth, after multivariable adjustment (AOR, 3.87; 95% CI, 1.22–12.31; *p* = 0.022). A similar pattern was observed between the ages of 2 and 3 years (AOR, 11.64; 95% CI, 2.03–66.62;* p* = 0.006). In contrast, among girls, no statistically significant associations were observed between a lack of outdoor play in both seasons and failure to achieve catch-up growth between the ages of 1 and 2 years (AOR, 1.90; 95% CI, 0.74–4.91; *p* = 0.185) or the ages of 2 and 3 years (AOR, 1.53; 95% CI, 0.30–7.80; *p* = 0.611). However, formal tests for interaction by sex were not statistically significant (*p* for interaction = 0.200 for ages 1–2 years;* p* = 0.718 for ages 2–3 years). Additional stratified analyses by gestational age (eTable 5) showed that the association between lack of outdoor play and failure to achieve catch-up growth remained statistically significant among term children but not among preterm children. In a sensitivity analysis restricted to singleton children (eTable 6), the results were consistent with the main analysis.

**Table 3:** Associations between outdoor play and catch-up growth, stratified by sex

Exposure	Not achieving catch-up growth			
	Boys		Girls	
	Multivariable		Multivariable	
	AOR (95% CI)	***p*** value	AOR (95% CI)	***p*** value
1–2 years				
Outdoor play in both summer and winter	1 [Reference]		1 [Reference]	
Outdoor play in either summer or winter	1.22 (0.59–2.55)	0.592	0.77 (0.44–1.33)	0.345
No outdoor play in either summer or winter	3.87 (1.22–12.31)	0.022^a^	1.81 (0.70–4.71)	0.222
2–3 years				
Outdoor play in both summer and winter	1 [Reference]		1 [Reference]	
Outdoor play in either summer or winter	0.62 (0.22–1.73)	0.361	1.18 (0.64–2.18)	0.594
No outdoor play in either summer or winter	11.64 (2.03–66.62)	0.006^a^	2.04 (0.39–10.78)	0.399

Odds ratios (ORs) and 95% confidence intervals (CIs) were estimated using logistic regression models stratified by sex

*Abbreviations*: *AOR* Adjusted odds ratio, *CI* Confidence interval

^a^*p* < 0.05

## Discussion

This large, nationwide birth cohort study showed that outdoor play between the ages of 1 and 2 years and 2 and 3 years was associated with catch-up growth in height by the age of 3 years among children born SGA. Specifically, the absence of outdoor play in both summer and winter between the ages of 1 and 2 years and 2 and 3 years was associated with a higher likelihood of failure to achieve catch-up growth. These associations were consistently observed after multivariable adjustment.

This study adds to the existing evidence on the relationship between outdoor play and catch-up growth among children born SGA, while also highlighting the potential influence of multiple covariates. The observed differences between univariate and multivariable estimates, particularly for outdoor play between ages 2 and 3 years, may further support the presence of confounding by multiple covariates rather than the influence of a single dominant factor.

In exploratory analyses, the association appeared to be more pronounced among boys than among girls, although no statistically significant interaction by sex was observed. In addition, the association was observed among term children but not among preterm children, which may reflect limited statistical power in the preterm subgroup. These subgroup findings should therefore be interpreted with caution.

Outdoor play behavior showed substantial consistency between ages 1–2 and 2–3 years. This suggests that outdoor play may reflect relatively stable lifestyle patterns during early childhood rather than transient exposures. Therefore, the observed associations may reflect not only the effects of outdoor play at specific ages but also the influence of sustained behavioral patterns over time.

Outdoor play is generally considered beneficial for healthy growth and development in children [27]. It is a major source of physical activity [28], and many national and international guidelines recommend a certain amount of physical activity for children [29, 30]. Physical activity is positively associated with multiple health indicators, including skeletal health, motor and cognitive development, and healthy body composition [31]. However, there is limited evidence focusing specifically on children born SGA. A previous study in adulthood reported no differences in physical activity levels or health-related quality of life associated with physical activity between individuals born SGA and those born appropriate for gestational age [15], suggesting that the long-term consequences of physical activity may differ from its role during early developmental periods. Few studies have investigated whether physical activity or outdoor play contributes to catch-up growth during early childhood in this population.

Several biological and behavioral mechanisms may underlie the observed associations. Previous studies showing the benefits of physical activity on skeletal development during childhood have suggested that outdoor play may contribute to bone and muscle growth through increased physical activity [32]. In addition, physical activity has been shown to increase circulating insulin-like growth factor-1, a key mediator of growth-plate chondrocyte proliferation and bone elongation [33]. Furthermore, sunlight exposure during outdoor play stimulates the endogenous production of vitamin D, which is primarily synthesized in the skin via ultraviolet radiation and is essential for skeletal growth [34]. Outdoor playtime has been associated with higher serum vitamin D levels and both summer and winter outdoor activities may influence vitamin D status [35–37].

Although vitamin D concentrations were not directly assessed in the present study, this pathway may be relevant for children born SGA, who are reported to have a higher risk of vitamin D deficiency compared with their appropriate-for-gestational-age peers [38]. In addition, previous studies have reported sex differences in vitamin D status during early childhood, with boys generally exhibiting higher serum concentrations compared with girls [39]. Behavioral differences may also contribute, given that boys have been reported to spend more time playing outdoors compared with girls at similar ages [27]. These biological and behavioral factors may partly account for the patterns observed in the present study, particularly in the sex-stratified analyses; however, this interpretation remains speculative and warrants further investigation.

The present findings have potential clinical and public health implications. Conventional postnatal health management for children born SGA has typically emphasized nutritional strategies such as breastfeeding in infancy and a balanced diet at later stages [6, 40], with few other universally recommended behavioral interventions. Our results suggest that encouraging outdoor play from early childhood may be a potentially relevant behavioral factor associated with linear growth in this high-risk population. However, outdoor play among children has declined in recent decades [16]. Despite increasing concerns about safety and environmental risks, outdoor play remains an essential component of early childhood development. Guidance on age-appropriate outdoor play may complement existing nutritional and medical strategies for children born SGA.

### Strengths and limitations

This study has several strengths, including its large, nationwide cohort, longitudinal design, and strict definition of SGA based on birth anthropometry and gestational age. The JECS recruited participants from geographically diverse regions of Japan, and previous reports indicate that key maternal and infant characteristics are broadly comparable with national population data, suggesting reasonable generalizability within Japan [18]. Nevertheless, differences in cultural contexts, environmental exposures, and childcare practices may limit the generalizability of these findings to other settings.

Several limitations should be acknowledged. First, many variables, including outdoor playtime, were assessed through caregiver-completed questionnaires, which may be subject to recall and reporting bias. In addition, the measures of outdoor playtime did not capture the intensity or quality of physical activity. Furthermore, outdoor playtime was dichotomized, which may have reduced the ability to detect a dose–response relationship. This approach was chosen to specifically identify children with no outdoor play, who may represent a high-risk group from a public health perspective. Second, serum vitamin D concentrations and other hormonal markers related to linear growth were not available for most participants, precluding direct evaluation of potential biological mediators. Previous developmental growth models suggest that nutritional intake and environmental exposures during early childhood may play an important role in linear growth compared with endocrine factors, although hormonal influences cannot be excluded [41]. Third, missing data may have introduced selection bias, particularly because the analyses were based on a complete-case approach. Although some differences were observed between included and excluded participants, no clear differences were observed in the distribution of the main exposure (outdoor play). However, substantial missingness in this variable among excluded participants warrants cautious interpretation of the potential for selection bias. Fourth, although we adjusted for a range of potential confounders, including socioeconomic indicators (maternal and paternal educational attainment and household income) and postnatal health conditions that may influence both outdoor play and growth (malignancy, cerebral palsy, and bronchial asthma), residual confounding by unmeasured or imperfectly measured factors, such as detailed parental health behaviors, aspects of the home environment, or underlying biological conditions, cannot be completely excluded. Fifth, reverse causation is possible, as children with poorer growth may be less likely to engage in outdoor play. Outdoor play was assessed prior to the evaluation of catch-up growth, which may reduce this concern to some extent. In addition, we adjusted for baseline body size at birth (length-for-age z-score) as well as postnatal health conditions that may influence both physical activity and growth (including malignancy, cerebral palsy, and bronchial asthma), which may partially account for pre-existing differences in growth status and health. Nevertheless, the possibility of reverse causation cannot be completely excluded. In addition, the wide confidence intervals observed in some subgroup analyses indicate substantial uncertainty in the estimates. These limitations should be considered when interpreting the findings.

## Conclusions

In conclusion, in this nationwide birth cohort, a lack of outdoor play during early childhood was associated with a higher likelihood of failure to achieve catch-up growth by the age of 3 years among children born SGA. Although the underlying biological and behavioral mechanisms remain to be clarified, these findings suggest that promoting outdoor play in early childhood may be a potentially modifiable factor associated with catch-up growth in early childhood for children born SGA. However, given the observational nature of the study, further investigation is needed to clarify causality and to determine whether promoting outdoor play can effectively support linear growth. Future studies incorporating objective measures of physical activity and relevant biomarkers are needed to further examine these associations and to explore the long-term effects of early outdoor play on growth and health outcomes.

## Supplementary Information

Supplementary Material 1: Supplementary tables for “Outdoor Play and Catch-up Growth in Small-for-Gestational-Age Children: A Nationwide Prospective Birth Cohort Study from the Japan Environment and Children’s Study”. This file contains Supplementary eTable 1 (comparison of baseline characteristics between included and excluded participants), Supplementary eTable 2 (baseline characteristics according to catch-up growth status at age 3 years), Supplementary eTable 3 (cross-tabulation of outdoor play patterns between ages 1–2 and 2–3 years), Supplementary eTable 4 (original questionnaire items on outdoor playtime used in the analysis), Supplementary eTable 5 (associations between outdoor play and catch-up growth stratified by gestational age), and Supplementary eTable 6 (sensitivity analysis restricted to singleton children).

## Data Availability

Data are unsuitable for public deposition due to ethical restrictions and legal framework of Japan. It is prohibited by the Act on the Protection of Personal Information (Act No. 57 of 30 May 2003, amendment on 9 September 2015) to publicly deposit the data containing personal information. Ethical Guidelines for Medical and Health Research Involving Human Subjects enforced by the Japan Ministry of Education, Culture, Sports, Science and Technology and the Ministry of Health, Labour and Welfare also restricts the open sharing of the epidemiologic data. All inquiries about access to data should be sent to: jecs-en@nies.go.jp. The person responsible for handling enquiries sent to this email address is Dr. Shoji F. Nakayama, JECS Programme Office, National Institute for Environmental Studies.

## Abbreviations

AOR: Adjusted odds ratio
CI: Confidence interval
IQR: Interquartile range
JECS: Japan Environment and Children’s Study
OR: Odds ratio
SGA: Small for gestational age
STROBE: Strengthening the Reporting of Observational Studies in Epidemiology
