# Supplementary material for: Outdoor play and catch-up growth in small-for-gestational-age children: a nationwide prospective birth cohort study from the Japan environment and children’s study
Source: BMC Public Health. 2026 Aug 3;26:3117. doi: 10.1186/s12889-026-28796-x (PMC13721432; doi:10.1186/s12889-026-28796-x)
Supplement: Supplementary file 1 [file 12889_2026_28796_MOESM1_ESM.docx]

**Supplementary Tables for:**

Outdoor Play and Catch-up Growth in Small-for-Gestational-Age Children: A Nationwide Prospective Birth Cohort Study from the Japan Environment and Children’s Study

Yoshinori Aoki, Michikazu Nakai, Hirotake Sawada, Hiroshi Moritake, and the Japan Environment and Children’s Study Group

**eTable 1. Comparison of baseline characteristics between included and excluded participants**

|  | **Participants** | |  |  |
| --- | --- | --- | --- | --- |
| **Characteristic** | **Included (n = 1,796)** | **Excluded (n =713)** | ***p* value** |  |
| Maternal age at delivery, median (IQR), y | 31.0 (28.0, 35.0) | 30.0 (26.0, 34.0) | <0.001 |  |
| Maternal height, median (IQR), cm | 157.0 (153.0, 160.0) | 156.0 (152.5, 160.0) | 0.005 |  |
| Maternal smoking during pregnancy |  |  | <0.001 |  |
| No | 1,661 (92.5%) | 575 (80.6%) |  |  |
| Yes | 115 (6.4%) | 107 (15.0%) |  |  |
| Missing data | 20 (1.1%) | 31 (4.3%) |  |  |
| Parity |  |  | <0.001 |  |
| 0 | 935 (52.1%) | 279 (39.1%) |  |  |
| ≥1 | 861 (47.9%) | 434 (60.9%) |  |  |
| Plurality |  |  | 0.93 |  |
| Singleton | 1,695 (94.4%) | 671 (94.1%) |  |  |
| Mutiple | 101 (5.6%) | 42 (5.9) |  |  |
| Mother educational level |  |  | <0.001 |  |
| Junior high school | 85 (4.7%) | 63 (8.8%) |  |  |
| High school | 571 (31.8%) | 252 (35.3%) |  |  |
| Technical college, Vocational school, Junior college | 739 (41.1%) | 280 (39.3%) |  |  |
| University/College, Graduate school | 373 (20.8%) | 78 (10.9%) |  |  |
| Missing data | 28 (1.6%) | 40 (5.6%) |  |  |
| Father educational level |  |  | <0.001 |  |
| Junior high school | 126 (7.0%) | 92 (12.9%) |  |  |
| High school | 639 (35.6%) | 302 (42.4%) |  |  |
| Technical college, Vocational school, Junior college | 432 (24.1%) | 129 (18.1%) |  |  |
| University/College, Graduate school | 562 (31.3%) | 146 (20.5%) |  |  |
| Missing data | 37 (2.1%) | 44 (6.2%) |  |  |
| Household annual income (JPY) |  |  | 0.002 |  |
| <2,000,000 | 85 (4.7%) | 56 (7.9%) |  |  |
| 2,000,000-3,999,999 | 607 (33.8%) | 244 (34.2%) |  |  |
| 4,000,000-5,999,999 | 537 (29.9%) | 170 (23.8%) |  |  |
| 6,000,000-7,999,999 | 257 (14.3%) | 82 (11.5%) |  |  |
| 8,000,000-9,999,999 | 122 (6.8%) | 28 (3.9%) |  |  |
| 10,000,000-11,999,999 | 35 (1.9%) | 8 (1.1%) |  |  |
| 12,000,000-14,999,999 | 13 (0.7%) | 4 (0.6%) |  |  |
| 15,000,000-19,999,999 | 9 (0.5%) | 3 (0.4%) |  |  |
| ≥20,000,000 | 5 (0.3%) | 2 (0.3%) |  |  |
| Missing data | 126 (7.0%) | 116 (16.3%) |  |  |
| Child's sex (boys) | 755 (42.0%) | 326 (45.7%) | 0.093 |  |
| Gestational age, median (IQR), wk | 39.0 (38.0, 40.0) | 39.0 (38.0, 40.0) | 0.76 |  |
| Preterm |  |  | 0.47 |  |
| No | 1,576 (87.8%) | 633 (88.8%) |  |  |
| Yes | 220 (12.2%) | 80 (11.2%) |  |  |
| Birth length, median (IQR), cm | 45.0 (43.6, 46.0) | 45.0 (44.0, 46.0) | 0.87 |  |
| Birth length z score, median (IQR) | -2.1 (-2.4, -1.5) | -2.1 (-2.3, -1.5) | 0.20 |  |
| Birth weight, median (IQR), g | 2372 (2126, 2575) | 2362.0 (2100, 2584) | 0.86 |  |
| Birth weight z score, median (IQR) | -2.1 (-2.4, -1.5) | -2.1 (-2.4, -1.5) | 0.20 |  |
| Feeding at 6 months of age |  |  | <0.001 |  |
| Breast milk only | 570 (31.7%) | 168 (23.6%) |  |  |
| Formula milk only | 49 (2.7%) | 19 (2.7%) |  |  |
| Mixed | 1147 (63.9%) | 356 (49.9%) |  |  |
| Missing data | 30 (1.7%) | 170 (23.8%) |  |  |
| Household smoking exposure |  |  | 0.007 |  |
| No | 1,071 (59.6%) | 467 (65.5%) |  |  |
| Yes | 725 (40.4%) | 246 (34.5%) |  |  |
| Postnatal health conditions | 179 (10.0%) | 29 (4.1%) | <0.001 |  |
| Outdoor play (1-2years) |  |  | 0.69 |  |
| Outdoor play in both summer and winter | 1,381 (76.9%) | 277 (38.8%) |  |  |
| Outdoor play in either summer or winter | 277 (15.4%) | 52 (7.3%) |  |  |
| No outdoor play in either summer or winter | 54 (3.0%) | 8 (1.1%) |  |  |
| Missing data | 84 (4.7%) | 376 (52.7%) |  |  |
| Outdoor play (2-3years) |  |  | 0.34 |  |
| Outdoor play in both summer and winter | 1,588 (88.4%) | 106 (14.9%) |  |  |
| Outdoor play in either summer or winter | 181 (10.1%) | 17 (2.4%) |  |  |
| No outdoor play in either summer or winter | 27 (1.5%) | 3 (0.4%) |  |  |
| Missing data | 0 | 587 (82.3%) |  |  |

Values are presented as number (percentage) for categorical variables and median (interquartile range) for continuous variables. Differences between included and excluded participants were assessed using the chi-square test for categorical variables and the Mann–Whitney U test for continuous variables.

**eTable 2. Baseline characteristics according to catch-up growth status at age 3 years**

|  | **Participants** |  |  |  |
| --- | --- | --- | --- | --- |
| **Characteristic** | **Total (n = 1,796)** | **Catch-up growth (n =1,500)** | **No catch-up growth (n=296)** | ***p* value** |
| Maternal age at delivery, median (IQR), y | 31.0 (28.0, 35.0) | 32.0 (28.0, 35.0) | 31.0 (27.0, 35.0) | 0.067 |
| Maternal height, median (IQR), cm | 157.0 (153.0, 160.0) | 157.0 (153.5, 161.0) | 154.0 (150.0, 158.0) | <0.001 |
| Maternal smoking during pregnancy |  |  |  | 0.12 |
| No | 1,661 (92.5%) | 1387 (92.4%) | 274 (92.5%) |  |
| Yes | 115 (6.4%) | 97 (6.5%) | 18 (6.1%) |  |
| Missing data | 20 (1.1%) | 16 (1.1%) | 4 (1.4%) |  |
| Parity |  |  |  | 0.055 |
| 0 | 935 (52.1%) | 796 (53.1%) | 139 (47.0%) |  |
| ≥1 | 861 (47.9%) | 704 (46.9%) | 157 (53.0%) |  |
| Plurality |  |  |  | 0.93 |
| Singleton | 1,695 (94.4%) | 1426 (95.1%) | 269 (90.9%) | <0.001 |
| Mutiple | 101 (5.6%) | 74 (4.9%) | 27 (9.1%) |  |
| Mother educational level |  |  |  | 0.008 |
| Junior high school | 85 (4.7%) | 61 (4.1%) | 24 (8.1%) |  |
| High school | 571 (31.8%) | 472 (31.5%) | 99 (33.4%) |  |
| Technical college, Vocational school, Junior college | 739 (41.1%) | 626 (41.7%) | 113 (38.2%) |  |
| University/College, Graduate school | 373 (20.8%) | 322 (21.5%) | 51 (17.2%) |  |
| Missing data | 28 (1.6%) | 19 (1.3%) | 9 (3.0%) |  |
| Father educational level |  |  |  | 0.48 |
| Junior high school | 126 (7.0%) | 105 (7.0%) | 21 (7.1%) |  |
| High school | 639 (35.6%) | 535 (35.7%) | 104 (35.1%) |  |
| Technical college, Vocational school, Junior college | 432 (24.1%) | 353 (23.5%) | 79 (26.7%) |  |
| University/College, Graduate school | 562 (31.3%) | 480 (32.0%) | 82 (27.7%) |  |
| Missing data | 37 (2.1%) | 27 (1.8%) | 10 (3.4%) |  |
| Household annual income (JPY) |  |  |  | 0.54 |
| <2,000,000 | 85 (4.7%) | 72 (4.8%) | 13 (4.4%) |  |
| 2,000,000-3,999,999 | 607 (33.8%) | 497 (33.1%) | 110 (37.2%) |  |
| 4,000,000-5,999,999 | 537 (29.9%) | 451 (30.1%) | 86 (29.1%) |  |
| 6,000,000-7,999,999 | 257 (14.3%) | 222 (14.8%) | 35 (11.8%) |  |
| 8,000,000-9,999,999 | 122 (6.8%) | 99 (6.6%) | 23 (7.8%) |  |
| 10,000,000-11,999,999 | 35 (1.9%) | 33 (2.2%) | 2 (0.7%) |  |
| 12,000,000-14,999,999 | 13 (0.7%) | 10 (0.7%) | 3 (1.0%) |  |
| 15,000,000-19,999,999 | 9 (0.5%) | 8 (0.5%) | 1 (0.3%) |  |
| ≥20,000,000 | 5 (0.3%) | 4 (0.3%) | 1 (0.3%) |  |
| Missing data | 126 (7.0%) | 104 (6.9%) | 22 (7.4%) |  |
| Child's sex (boys) | 755 (42.0%) | 635 (42.3%) | 120 (40.5%) | 0.568 |
| Gestational age, median (IQR), wk | 39.0 (38.0, 40.0) | 39.0 (38.0, 40.0) | 39.0 (37.0, 40.0) | <0.001 |
| Preterm |  |  |  | <0.001 |
| No | 1,576 (87.8%) | 1340 (89.3%) | 236 (79.7%) |  |
| Yes | 220 (12.2%) | 160 (10.7%) | 60 (20.3%) |  |
| Birth length, median (IQR), cm | 45.0 (43.6, 46.0) | 45.0 (44.0, 46.2) | 44.5 (42.8, 45.7) | <0.001 |
| Birth length z score, median (IQR) | -2.1 (-2.4, -1.5) | -2.1 (-2.4, -1.4) | -2.1 (-2.5, -1.7) | <0.001 |
| Birth weight, median (IQR), g | 2372 (2126, 2575) | 2386.0 (2164, 2586) | 2283.0 (1915, 2515) | <0.001 |
| Birth weight z score, median (IQR) | -2.1 (-2.4, -1.5) | -2.1 (-2.4, -1.4) | -2.1 (-2.5, -1.7) | <0.001 |
| Feeding at 6 months of age |  |  |  | 0.74 |
| Breast milk only | 570 (31.7%) | 482 (32.1%) | 88 (29.7%) |  |
| Formula milk only | 49 (2.7%) | 42 (2.8%) | 7 (2.4%) |  |
| Mixed | 1147 (63.9%) | 950 (63.3%) | 197 (66.6%) |  |
| Missing data | 30 (1.7%) | 26 (1.7%) | 4 (1.4%) |  |
| Household smoking exposure |  |  |  | 0.14 |
| No | 1,071 (59.6%) | 906 (60.4%) | 165 (55.7%) |  |
| Yes | 725 (40.4%) | 594 (39.6%) | 131 (44.3%) |  |
| Postnatal health conditions | 179 (10.0%) | 143 (9.5%) | 36 (12.2%) | 0.17 |
| Outdoor play (1-2years) |  |  |  | 0.01 |
| Outdoor play in both summer and winter | 1,381 (76.9%) | 1152 (76.8%) | 229 (77.4%) |  |
| Outdoor play in either summer or winter | 277 (15.4%) | 236 (15.7%) | 41 (13.9%) |  |
| No outdoor play in either summer or winter | 54 (3.0%) | 37 (2.5%) | 17 (5.7%) |  |
| Missing data | 84 (4.7%) | 75 (5.0%) | 9 (3.0%) |  |
| Outdoor play (2-3years) |  |  |  | 0.14 |
| Outdoor play in both summer and winter | 1,588 (88.4%) | 1333 (88.9%) | 255 (86.1%) |  |
| Outdoor play in either summer or winter | 181 (10.1%) | 148 (9.9%) | 33 (11.1%) |  |
| No outdoor play in either summer or winter | 27 (1.5%) | 19 (1.3%) | 8 (2.7%) |  |

Values are presented as number (percentage) for categorical variables and median (interquartile range) for continuous variables. Catch-up growth was defined as achieving a height above −2 standard deviation scores at age 3 years. Differences between groups were assessed using the chi-square test for categorical variables and the Mann–Whitney U test for continuous variables.

**eTable 3. Cross-tabulation of outdoor play patterns between ages 1–2 years and 2–3 years**

|  | **Outdoor play at ages 2-3 years** | |  |
| --- | --- | --- | --- |
| **Outdoor play at ages 1-2 years** | **Not consistent outdoor play (none or either season)** | **Consistent outdoor play (both summer and winter)** | **Total** |
| **Not consistent outdoor play (none or either season)** | 122 (7.1%) | 75 (4.4%) | 197 (11.5%) |
| **Consistent outdoor play (both summer and winter)** | 209 (12.2%) | 1306 (76.3%) | 1515 (88.5%) |
| **Total** | 331 (19.3%) | 1381 (80.7%) | 1712 (100%) |

Values are presented as number (percentage). Outdoor play was originally assessed separately for summer and winter and categorized into five levels; for this analysis, it was grouped into “consistent outdoor play” (outdoor play in both summer and winter) and “not consistent outdoor play” (no outdoor play or outdoor play in either season). The association between outdoor play patterns at ages 1–2 and 2–3 years was evaluated using Pearson’s chi-square test (χ² = 258.97, p < 0.001).

**eTable 4. Original questionnaire items on outdoor playtime used in the analysis**

| **Exposure** | **Total (n = 1,796)** | **Boys (n = 755)** | **Girls (n = 1,041)** |
| --- | --- | --- | --- |
| **Summer (1–2 years)** |  |  |  |
| None | 78 (4.3%) | 36 (4.8%) | 42 (4.0%) |
| <1 h | 569 (31.7%) | 237 (31.4%) | 332 (31.9%) |
| 1–3 h | 998 (55.6%) | 407 (53.9%) | 591 (56.8%) |
| >3 h | 74 (4.1%) | 39 (5.2%) | 35 (3.4%) |
| Unknown | 25 (1.4%) | 7 (0.9%) | 18 (1.7%) |
| Not available | 52 (2.9%) | 29 (3.8%) | 23 (2.2%) |
| **Winter (1–2 years)** |  |  |  |
| None | 311 (17.3%) | 124 (16.4%) | 187 (18.0%) |
| <1 h | 865 (48.2%) | 347 (46.0%) | 518 (49.8%) |
| 1–3 h | 509 (28.3%) | 233 (30.9%) | 276 (26.5%) |
| >3 h | 32 (1.8%) | 14 (1.9%) | 18 (1.7%) |
| Unknown | 26 (1.4%) | 7 (0.9%) | 19 (1.8%) |
| Not available | 53 (3.0%) | 30 (4.0%) | 23 (2.2%) |
| **Summer (2–3 years)** |  |  |  |
| None | 32 (1.8%) | 11 (1.5%) | 21 (2.0%) |
| <1 h | 438 (24.4%) | 172 (22.8%) | 266 (25.6%) |
| 1–3 h | 1,184 (65.9%) | 504 (66.8%) | 680 (65.3%) |
| >3 h | 142 (7.9%) | 68 (9.0%) | 74 (7.1%) |
| **Winter (2–3 years)** |  |  |  |
| None | 203 (11.3%) | 82 (10.9%) | 121 (11.6%) |
| <1 h | 802 (44.7%) | 332 (44.0%) | 470 (45.1%) |
| 1–3 h | 735 (40.9%) | 307 (40.7%) | 428 (41.1%) |
| >3 h | 56 (3.1%) | 34 (4.5%) | 22 (2.1%) |

The questionnaire was completed by caregivers. Outdoor playtime was assessed separately for summer and winter using predefined response categories. These responses were used to derive exposure variables for the main analyses.

**eTable 5. Associations between outdoor play and catch-up growth stratified by gestational age**

|  | **Not achieving catch-up growth** | | |  |
| --- | --- | --- | --- | --- |
|  | **Term** |  | **Pretrm** |  |
|  | **Multivariable** |  | **Multivariable** |  |
| **Exposure** | **AOR (95% CI)** | ***p* value** | **AOR (95% CI)** | ***p* value** |
| **1–2 years** |  |  |  |  |
| Outdoor play in both summer and winter | 1 [Reference] |  | 1 [Reference] |  |
| Outdoor play in either summer or winter | 0.92 (0.57-1.48) | 0.720 | 0.97 (0.32-2.91) | 0.959 |
| No outdoor play in either summer or winter | 2.60 (1.17-5.77) | 0.019 | 1.42 (0.21-9.66) | 0.719 |
| **2–3 years** |  |  |  |  |
| Outdoor play in both summer and winter | 1 [Reference] |  | 1 [Reference] |  |
| Outdoor play in either summer or winter | 0.97 (0.55-1.70) | 0.907 | 0.91 (0.22-3.70) | 0.890 |
| No outdoor play in either summer or winter | 5.66 (1.67-19.15) | 0.005 | 0.71 (0.03-14.33) | 0.821 |

Odds ratios (ORs) and 95% confidence intervals (CIs) were estimated using logistic regression models stratified by gestational age (preterm and term)..

**eTable 6. Sensitivity analysis restricted to singleton children**

|  | **Not achieving catch-up growth** | |
| --- | --- | --- |
|  | **Multivariable** |  |
| **Exposure** | **AOR (95% CI)** | ***p* value** |
| **1–2 years** |  |  |
| Outdoor play in both summer and winter | 1 [Reference] |  |
| Outdoor play in either summer or winter | 0.97 (0.62-1.52) | 0.894 |
| No outdoor play in either summer or winter | 2.79 (1.28-6.11) | 0.010 |
| **2–3 years** |  |  |
| Outdoor play in both summer and winter | 1 [Reference] |  |
| Outdoor play in either summer or winter | 0.88 (0.51-1.53) | 0.660 |
| No outdoor play in either summer or winter | 5.02 (1.60-15.79) | 0.006 |

Odds ratios (ORs) and 95% confidence intervals (CIs) were estimated using logistic regression models among singleton children.
